# Meeting report on FASEB protein arginine methylation: mechanism to therapeutics

**DOI:** 10.1016/j.jbc.2026.111448

**Published:** 2026-04-11

**Authors:** David Shechter, Rong Huang, Pooja Shah, Thierry Dubois, Xiao-Meng Li, Andrey Parhitko, Mark T. Bedford, Shihuan Kuang, Taiping Chen, Yanzhong Yang, Purusharth Rajyaguru, Dalia Barsyte-Lovejoy, Xinyang Zhao, Wei Xu, Clare C. Davies, Irina Tikhanovich, Ngoc Tung Tran, Koichiro Kako, Misuzu Hashimoto, Minkui Luo, Joan M. Hevel, Akiyoshi Fukamizu, Michael C. Yu

**Affiliations:** 1 Department of Biochemistry, Albert Einstein College of Medicine, Bronx, New York, USA; 2 Borch Department of Medicinal Chemistry and Molecular Pharmacology, Purdue University, West Lafayette, Indiana, USA; 3 Department of Investigational Cancer Therapeutics, The University of Texas MD Anderson Cancer Center, Houston, Texas, USA; 4 Institut Curie, PSL University, Sorbonne University, Translational Research Department & CNRS UMR144, Paris, France; 5 The Hong Kong University of Science and Technology (Guangzhou), Guangzhou, China; 6 Aging Institute, UPMC and University of Pittsburgh, Pittsburgh, Pennsylvania, USA; 7 Department of Epigenetics and Molecular Carcinogenesis, The University of Texas MD Anderson Cancer Center, Houston, Texas, USA; 8 Duke University School of Medicine, Durham, North Carolina, USA; 9 Department of Cancer Genetics and Epigenetics, Beckman Research Institute, City of Hope Cancer Center, Duarte, California, USA; 10 Indian Institute of Science, Bengaluru, Karnataka, India; 11 Department of Pharmacology and Toxicology, University of Toronto and Structural Genomics Consortium, University Health Network, Toronto, ON, Canada; 12 Department of Pathology and Laboratory Medicine, University of Kansas Medical Center, Kansas City, Kansas, USA; 13 McArdle Laboratory for Cancer Research, University of Wisconsin–Madison, Madison, Wisconsin, USA; 14 Department of Cancer and Genomic Sciences, University of Birmingham, Birmingham, UK; 15 Department of Internal Medicine and Liver Center, University of Kansas Medical Center, Kansas City, Kansas, USA; 16 Herman B Wells Center for Pediatric Research, Department of Pediatrics, Indiana University School of Medicine, Indianapolis, Indiana, USA; 17 Life Science Center for Survival Dynamics, Tsukuba Advanced Research Alliance (TARA), University of Tsukuba, Tsukuba, Japan; 18 Laboratory of Biological Chemistry, Faculty of Applied Biological Sciences, Gifu University, Gifu, Japan; 19 Chemical Biology Program, Memorial Sloan Kettering Cancer Cente, New York, USA; 20 Department of Pharmacology, Weill Cornell Medical College, Cornell University, New York, New York, USA; 21 Department of Chemistry and Biochemistry, Utah State University, Logan, Utah, USA; 22 Department of Biological Sciences, University at Buffalo, Buffalo, New York, USA

**Keywords:** cancer therapeutics, chemical probes, genome stability, methylarginine, methylarginine reader proteins, protein arginine methylation, PRMTs, RNA processing

## Abstract

The inaugural FASEB conference *Protein Arginine Methylation: Mechanism to Therapeutics* was held in Tsukuba, Japan (January 5–8, 2026), and brought together investigators studying protein arginine methyltransferases (PRMTs) and its methylarginine product. Post-translational arginine methylation, found as monomethylarginine (MMA/Rme1), asymmetric dimethylarginine (ADMA/Rme2a), and symmetric dimethylarginine (SDMA/Rme2s), is increasingly recognized as a central regulator of RNA metabolism, chromatin function, and cellular stress responses. Presentations highlighted emerging directions for the field, including mechanisms linking PRMT activity to RNA processing and gene expression, roles in genome stability, identification of new methylarginine reader proteins, and connections between PRMT function and cellular metabolism. Work on immune and antiviral pathways and neuroscience further expanded the biological scope of PRMT activity. At the same time, significant advances in chemical probes, degraders, and selective inhibitors are enabling more precise interrogation of PRMT biology and accelerating efforts to translate these discoveries into therapeutic strategies.

The inaugural in-person conference on Protein Arginine Methylation: Mechanism to Therapeutics was held in January 2026 in Tsukuba, Japan. Arginine methylation, a post-translational modification in eukaryotes occurring primarily as mono- (MMA/Rme1), asymmetric (ADMA/Rme2a), and symmetric (SDMA/Rme2s) dimethylarginine, is highly abundant but still only beginning to be understood (Fig. 1, *A* and *B*). At this FASEB conference, organized by Michael C. Yu, Akiyoshi Fukamizu, and^,^ Ho Man Chan, investigators shared new studies on protein arginine methyltransferases (PRMTs) and their cellular consequences that converged on six major themes: regulation of RNA processing and gene expression; maintenance of genome stability; expansion of the methylarginine reader repertoire; metabolism-methylation crosstalk; immune and antiviral roles by PRMTs; and advances in chemical biology and therapeutic development in oncology and beyond (Fig. 2). Taken together, these themes paint a picture of a field entering a mechanistically rich and therapeutically actionable era. Below, we highlight some key insights and emerging opportunities discussed at the conference.

**Figure 1:**
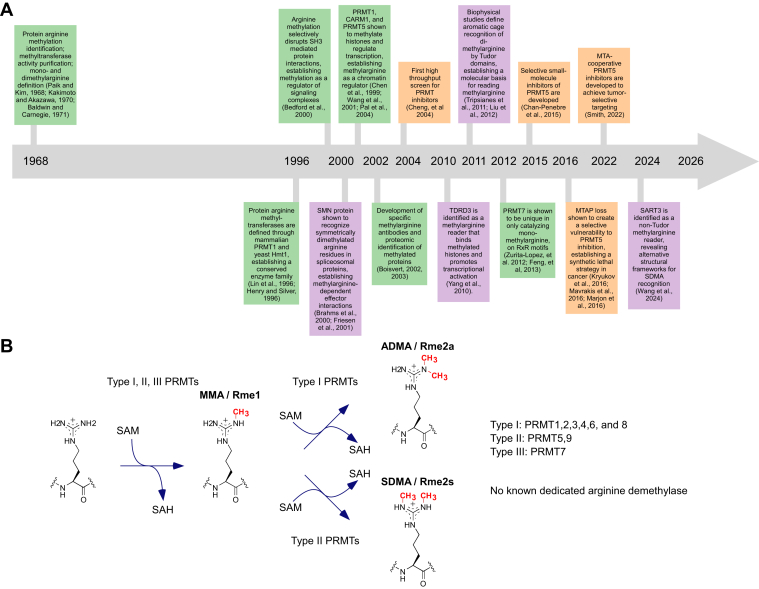
Protein arginine methylation from biochemical discovery to therapeutic targeting. *A*, Timeline of key advances in arginine methylation biology, highlighting the progression from early biochemical identification of methylarginine and methyltransferase activity (15, 16, 17) to molecular definition of protein arginine methyltransferases (PRMTs) (18, 19). Early mechanistic studies demonstrated that arginine methylation regulates protein–protein interactions (20) and chromatin function through histone methylation by CARM1/PRMT4, PRMT1, and PRMT5 (21, 22, 23), while PRMT7 is unique in only catalyzing monomethylation (24, 25). Tool development of specific antibodies (26, 27) and inhibitors helped advance the field. The identification of methylarginine reader proteins, including SMN and TDRD3, and the structural definition of Tudor domain recognition established principles of methylarginine-dependent signaling (28, 29, 30, 31, 32, 33). Translational advances include the development of selective PRMT5 inhibitors (34, 35) and the discovery that MTAP loss leads to accumulation of methylthioadenosine (MTA), creating a synthetic lethal vulnerability to PRMT5 inhibition (36, 37, 38). More recent discoveries expanded methylarginine reader biology beyond Tudor domains, including SART3 (39) and established MTA-cooperative PRMT5 inhibitors that exploit tumor metabolic state for selective targeting (40, 41). Colored boxes denote conceptual categories: enzyme discovery, biochemistry, and biology (green); methylarginine readers and mechanisms (purple); and therapeutic development (orange). *B*, Biochemical reactions catalyzed by PRMTs. Type I, II, and III PRMTs catalyze processive methylation of arginine residues using S-adenosylmethionine (SAM) as a methyl donor, generating monomethylarginine (MMA/Rme1), asymmetric dimethylarginine (ADMA/Rme2a), or symmetric dimethylarginine (SDMA/Rme2s), with S-adenosylhomocysteine (SAH) as a byproduct. PRMT family members are classified by product specificity: Type I catalyzing MMA and ADMA (PRMT1, 2, 3, 4, 6, and 8), Type II catalyzing MMA and SDMA (PRMT5 and 9), and Type III only catalyzing MMA (PRMT7).

**Figure 2:**
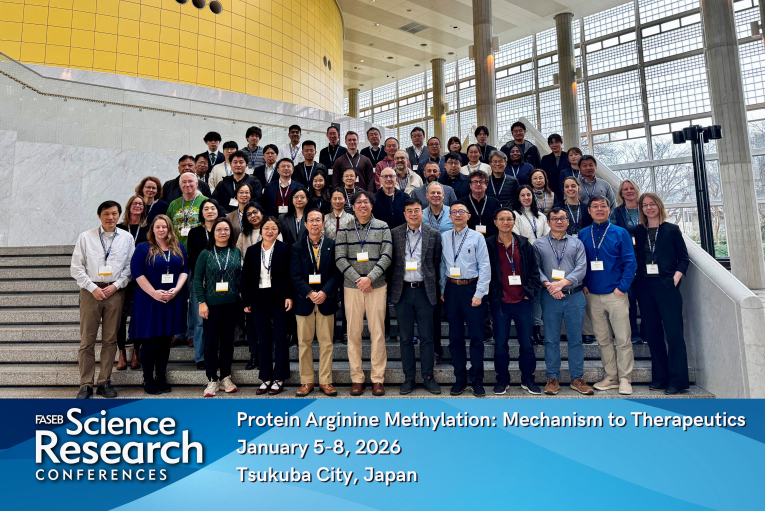
Photograph of meeting attendees.

## PRMTs as master regulators of RNA-based gene expression

A dominant theme throughout the meeting was the central role of arginine methylation in RNA-mediated cellular processes, spanning transcription, splicing, RNA trafficking, 3′ end formation, and ribonucleoprotein (RNP) assembly. Several talks shared how PRMTs function to regulate splicing. Wenjian Gan identified PRPF6 as a PRMT5 substrate whose methylation at R23 enables binding to TXNL4A, establishing a methylation-dependent splicing module that controls DNA repair transcripts. Jian Xu showed that PRMT1-SFPQ complexes regulate intron retention and nuclear mRNA storage in craniofacial development, while Takehiro Yamamoto linked PRMT-mediated splicing programs to cancer cell identity and immune signaling pathways. The meeting also highlighted PRMT-dependent RNA localization and transport in neurons. Kwok-On Lai demonstrated how arginine methylation coordinates kinesin-based transport of translationally active RNPs in synapses. Ofri Abraham from Mike Fainzilber’s group showed that nucleolin GAR-domain methylation may participate in neuronal RNA transport and axonal length sensing. Koichiro Kako, from Akiyoshi Fukamizu’s group, identified stomatin-1 as a novel monomethylated protein, implicating a role for this modification in ion channel regulation (1). Purusharth Rajyaguru shared his group’s work on a catalytic-activity independent role of yeast Hmt1 and its mammalian homolog, PRMT1, in promoting the disassembly of synuclein aggregates and facilitating their degradation, thereby modulating synuclein toxicity (2).

An exciting new direction was PRMT-dependent processing of histone mRNAs, underscored by two talks: David Shechter’s presentation showing that PRMT5 sustains histone gene expression through S-phase likely through H4R3me2s methylation (3); this theme was reinforced by Xiao-Meng Li, who identified ASCC2/3 as a specific H3R2me2s reader that localizes to histone gene clusters with NPAT and regulates histone mRNA 3′ end processing. These findings position PRMT5-catalyzed symmetric dimethylarginine (Rme2s/SDMA) recognition at the core of histone mRNA biogenesis.

Together, these studies establish methylarginine as a key coordinator of RNA fate across splicing, transport, processing, and nuclear organization. Important open questions remain regarding how these events are integrated with signaling pathways and cellular metabolism.

## Genome stability as a unifying output of PRMT function

Across model systems, PRMTs emerged as key guardians of genome integrity, a conclusion supported from replication, repair, and chromatin perspectives. Michael Ferguson presented compelling data on MCM4 arginine methylation, demonstrating its contribution to replication fork stability. Ernesto Guccione’s mechanistic study connected PRMT5 directly to telomerase trafficking, TPP1 splicing, Cajal body integrity, and telomere elongation, thereby revealing a selective vulnerability in TERT-high cancers. Clare Davies similarly showed that RUVBL1-R205 methylation is required for homologous recombination, predicts sensitivity to PRMT5 inhibitors and DNA-damaging agents, and integrates PRMT5 activity with the E3 ligase FBXO4 after DNA damage. This work highlighted a major knowledge gap in how PRMTs achieve temporal and spatial substrate specificity. David Shechter further demonstrated that loss of PRMT5-dependent histone gene regulation directly led to p53-independent micronuclei and genome instability.

The role of methylation in R-loop homeostasis was reinforced by Stephane Richard’s keynote address, who highlighted earlier studies of PRMT5-and DDX5-dependent suppression of R-loops at transcription start and termination sites, linking RNA helicase function to methylarginine code. This theme was broadened to neurodegenerative disease through Kensuke Ikenaka’s ALS findings, in which dysregulated PRMT1-dependent methylation correlated with DNA damage signatures and respiratory-associated upregulation of PRMT1.

As genome maintenance emerged as a unifying phenotype of disrupted methylation-dependent RNA processing and chromatin assembly, PRMTs are now central genome stability factors. Future studies in DNA repair and neurodegenerative diseases will therefore likely uncover important new biology and opportunities for therapeutic intervention.

## Reader biology: Expansion of the SDMA-recognition landscape

A highlight of the meeting was the expansion of methylarginine reader biology. In his keynote, Mark Bedford synthesized 3 decades of work, including SND1 as a major effector of SDMA-driven transcriptional programs in cancer. Stephane Richard, in his keynote also emphasized this principle more broadly, noting conserved aromatic cages in multiple helicases and RNA-binding proteins. Yanzhong Frankie Yang showed how the TDRD3 methylarginine reader maintains active chromatin through facilitation of FACT histone chaperone recruitment, and how this epigenetic regulation is controlled by a negative crosstalk between arginine methylation and phosphorylation. Thierry Dubois demonstrated that ESCRT component ALIX methylation by CARM1 disrupts binding of SH3 domain-containing partners and, in turn, regulates cytokinesis and potentially other ESCRT-dependent processes (4). Xiao-Meng Li’s integrated chemoproteomic strategy combining photo-crosslinking, bioorthogonal chemistry, and quantitative proteomics identified ASCC2/3 as specific H3R2me2s readers, one of the diverse non-Tudor readers, and such finding expanded the arginine methylation reader landscape. Ernesto Guccione identified WDR79 (also known as TCAB and WRAP53) as a putative Rme2s reader. These discoveries marked a turning point as the field is now identifying specialized reader complexes that decode SDMA marks with highly selective biological outcomes. The meeting made clear that reader discovery is now accelerating, reshaping the map of methylarginine signaling as bromodomain and chromodomain proteins did for lysine-acetyl and lysine-methyl pathways. Future directions for the field include more rigorous and systematic identification of reader domains and also new avenues, including how methylarginine may influence protein: nucleic acid interactions.

## Metabolism - methylation coupling: PRMTs as sensors and integrators of cellular state

Multiple speakers demonstrated that PRMTs interface deeply with metabolic pathways, regulating nutrient sensing, mitochondrial function, and stress adaptation. Shihuan Kuang opened the conference with the surprising discovery of a physiological role for PRMT5 in lipid metabolism and lipid droplet homeostasis in skeletal muscle and adipose tissue. Michael Yu and Akiyoshi Fukamizu both further connected PRMT activity with metabolic rewiring, demonstrating systemic PRMT1-dependent regulation of energy balance, stress responses, and aging. Michale Yu showed that methylation of an RNA-binding protein by yeast PRMT1-homologue promotes yeast metabolic reprogramming when transitioning from fermentative to respiratory growth. Akiyoshi Fukamizu showed that a single amino acid change (H179Y) makes human PRMT1 more catalytically active than mouse PRMT1 by strengthening substrate–SAM interactions, and humanized PRMT1 knock-in mice were generated to test the effects of this enhanced activity *in vivo*, demonstrating that increased PRMT1 activity supports organismal stress adaptation. Takahiro Yamamoto provided evidence that PRMTs drive glucose metabolic shifts during cancer progression, while Irina Tikhanovich presented work showing that hepatic PRMT6 is downregulated in alcohol-associated liver disease and inversely correlates with fibrosis severity. PRMT6 acts primarily in liver macrophages and endothelial cells to restrain pro-fibrotic signaling. To identify downstream effectors that mediate the beneficial effects of methionine restriction, Andrey Parkhitko investigated the relationship between dietary methionine levels, downstream methyltransferases, and organismal stress resilience in *Drosophila melanogaster*. His lab performed a genetic screen targeting all predicted methyltransferases encoded in the *Drosophila* genome and identified multiple PRMTs that phenocopy the effects of methionine restriction. He further showed that these effects are conserved across flies, yeast, and human cells. These exciting new observations establish arginine methylation as a nutrient-responsive biochemical network that adjusts cell physiology in response to stress. Xinyang Zhao further reported that upregulation of PRMT1 switches the megakaryoctyes to rely on glycolysis (5) and that PRMT1 renders platelets hyperactive in response to thrombin and other agonists. Upregulation of PRMT1 in platelets was also observed in a diabetic mouse model. Fabao Liu identified ALIX as a CARM1 substrate and revealed critical role of CARM1-ALIX in exosome biosynthesis and controlling hypoxanthine levels in activated CD8+ T cells. These findings establish the CARM1-ALIX-hypoxanthine axis as an immunosuppressive mechanism (6). Future avenues will include understanding how PRMTs are responsive—through SAM/SAH levels, by signaling pathways, or some other mechanisms—and how these pathways may be therapeutically targeted.

## Immune and antiviral pathways: PRMTs as regulators of host defense

A major frontier reflected in the meeting was the widespread influence of PRMTs on the immune system and antiviral signaling. Stephane Richard in his keynote highlighted roles for PRMT7 in dsDNA sensing, antiviral programs, and interferon activation. He further showed exciting work on PRMT7 regulating memory T-cells, suggesting that PRMT7 chemical inhibition may allow their expansion and improve CAR-T therapy. Taiping Chen reported that PRMT6 antagonizes DNA methylation by preventing UHRF1 chromatin association, underscoring the finding on crosstalk between arginine methylation and other oncogenic pathways. Wei Xu also addressed these concepts in cancer immunology, showing CARM1-controlled regulation of CD8+ T cell evasion in triple-negative breast cancer (TNBC). Furthermore, Wei Xu identified MAP2K4 as a CARM1 substrate in TNBCs. Inhibiting CARM1-mediated MAP2K4 methylation leads to AKT activation, supporting a combination of CARM1 inhibition and PI3K inhibitors in TNBC therapy (7). Xinyang Zhao expanded the immunological footprint of PRMT1, showing its requirement for inflammation-triggered megakaryopoiesis. Interestingly, PRMT1 is required for the antigen-presenting activity of the immune megakaryocytes. In addition, Misuzu Hashimoto linked PRMT1 to CNS development and presented a model in which PRMT1 coordinates cell-type specific pathways critical for brain maturation. Work from Dalia Barsyte-Lovejoy demonstrated a new chemical probe that inhibits PRMT9 and PRMT5, expanding the toolkit for studying symmetric arginine methylation. In macrophages, this chemical probe altered the splicing of inflammatory regulators and modulated oxidative stress responses, highlighting PRMT-linked pathway in immune cell function. Across these talks, PRMTs consistently appeared as gatekeepers of inflammation, antiviral defense, and T cell fitness, positioning methylarginine signaling as a therapeutic interface between cancer, infection, and immunity.

## Chemical tools and therapeutic innovation: A maturing translational pipeline

Encouraged by the remarkable discovery a decade ago of true cancer-targeting synthetic lethality of PRMT5 loss or inhibition with the *MTAP* deletion found in at least 15% of human solid tumors, the meeting showcased an unprecedented wave of chemical biology and drug development. The clinical development of PRMT5 inhibitors has shifted from first generation pan-PRMT5 inhibitors, limited by toxicity, to second-generation MTA (accumulated metabolite found in MTAP-deficient cells)-cooperative agents that selectively target MTAP-loss tumors which are in ∼15% of cancers by exploiting synthetic lethality (8). Early clinical data show broad promise with many Phase II trials currently enrolling patients. Ho Man Chan, Han Xu, Pooja Shah, Haiping Wu, and Haiyan Ying presented data on a pipeline of these compounds, from AZD3470 and ABSK131 to MTA-cooperative PRMT5 degraders. Ho Man Chan and other speakers highlighted the new discovery of *MTAP*-silenced, but not deleted, tumors, suggesting that more cancers may be amenable to second-generation PRMT5 inhibition. Kathleen Mulvaney leveraged the second-generation inhibitors in CRISPR screens to identify new synthetic pathways for even more opportunity for combination therapy (9). As a part of his keynote presentation, Mark Bedford presented an MTA-cooperative strategy for PRMT1, inspired by the clinical progress of MTA-cooperative PRMT5 inhibitors and the emerging issue of intrinsic/acquired resistance despite persistently high MTA in resistant tumors. The conference was abuzz with enthusiasm for clinical-trial success of these compounds, especially for currently untreatable but frequently *MTAP*-deleted cancers like GBM and pancreatic cancer.

Minkui Luo summarized a decade of innovation enabling cofactor-engineered BPPM profiling, clickable SAM analogs, covalent trapping of methyltransferases, and next-generation active-site mimics (10). Wei Xu described the development of a CARM1 PROTAC based on the chemical structure of CARM1 inhibitor, TP-064 (11). Rong Huang presented the development of a focused library to probe subtle differences in the PRMT active site, employing a hybrid design strategy that integrates linker length optimization and fully engagement of the active site to discover potent and isoform-selective inhibitors (12). Ngoc Tung Tran identified a novel vulnerability of PRMT1 in multiple myeloma using both cellular systems and xenograft mouse models (13). He also reviewed the emerging roles of PRMTs in multiple myeloma and proposed an innovative approach to target PRMTs specifically in multiple myeloma (14). This approach utilizes a BCMA antibody-conjugated lipid nanoparticle platform to enable efficient and selective knockdown of target genes in multiple myeloma cell lines.

The field is now equipped with selective inhibitors and degraders, forming a sophisticated toolkit for both mechanism and medicine. New opportunities will include more specific targeting of all nine PRMTs, perhaps through unique pockets or sites found outside the catalytic site, and sites that take advantage of negative allostery as shown by the Hevel group’s characterization of half-of-sites reactivity in PRMT1, to further separate function and allow more targeted interventions.

## Conclusion

Taken together, the 2026 FASEB PRMT meeting showcased a field that has uncovered important basic biological mechanisms and expanded therapeutic opportunities. PRMTs have emerged as master regulators of RNA programs, guardians of genome stability, read by methylarginine reader proteins, and interconnected with metabolism, immunity, and stress responses. Technological and therapeutic innovation is now accelerating rapidly, positioning arginine methylation as a frontier with both mechanistic depth and translational potential. The meeting established the foundation for a cohesive research community and set a clear trajectory for future discovery in methylarginine biology.

## Conflict of interest

The author declares that they have no conflicts of interest with the contents of this article.

## References

[1] Uetake T., Kako K., Daitoku H., Fukamizu A. (2026). Identification of stomatin-1 (STO-1) as a novel arginine monomethylated protein in Caenorhabditis elegans. Biosci. Biotechnol. Biochem..

[2] Dewasthale S., Rajyaguru P.I. (2026). PRMT1/Hmt1 drives α-synuclein aggregate dissolution through a catalysis-independent pathway. bioRxiv.

[3] Roth J.S., DeAngelo J.D., Young D.L., Maron M.I., Saha A., Pinto H. (2025). PRMT5 Activity Sustains Histone Production to Maintain Genome Integrity. bioRxiv.

[4] Huard S., Suresh S., Vayr J., Ruggiero S., Dakroub R., Masson V. (2025). CARM1-mediated Methylation Controls Interactions of ALIX with Key Partners Important for Cytokinesis. bioRxiv.

[5] Su H., Sun Y., Guo H., Sun C.-W., Chen Q., Liu S. (2025). PRMT1-mediated metabolic reprogramming promotes leukemogenesis. eLife.

[6] Yin J., Su Z., Hu X., Sun H., Sun Z., Zhou S. (2026). CARM1-mediated hypoxanthine-enriched exosomes rewire inosine metabolism and impair CD8(+) T cell antitumor function. Cell Death Differ..

[7] Xie H., Bacabac M.S., Ma M., Kim E.J., Wang Y., Wu W. (2023). Development of potent and selective coactivator-associated arginine methyltransferase 1 (CARM1) degraders. J. Med. Chem..

[8] Rodon J., Johnson M.L., George B., Shah P.A., Arbour K.C. (2026). MTAP deletion in oncogenesis: a synthetic lethality scenario. Cancer Res..

[9] Knoll N., Masser S., Bordas B., Ebright R.Y., Li G., Kesar D. (2025). CRISPR–drug combinatorial screening identifies effective combination treatments for MTAP-deleted cancer. Cancer Res..

[10] Zong Y., Zhang N., Lin J., Li Z., Zou Y., Chaudhuri R. (2026). Assembling ternary dead-end complex for covalent trapping of protein lysine methyltransferases. J. Am. Chem. Soc..

[11] Kim E.J., Wang Y., Chen Y.L., Ma M., Liu P., Bacabac M.S. (2025). CARM1-Mediated MAP2K4 methylation potentiates the oncogenic functions of MAP2K4 and constitutes a targetable dependency in triple-negative breast cancer. Cancer Res..

[12] Deng Y., Kim E.-J., Song X., Kulkarni A.S., Zhu R.X., Wang Y. (2024). An adenosine analogue library reveals insights into active sites of protein arginine methyltransferases and enables the discovery of a selective PRMT4 inhibitor. J. Med. Chem..

[13] Nguyen H.P., Le A.Q., Liu E., Cesarano A., DiMeo F., Perna F. (2023). Protein arginine methyltransferase 1 is a therapeutic vulnerability in multiple myeloma. Front. Immunol..

[14] Qaddoura S.F., Liu E., Walker B.A., Tran N.T. (2025). Emerging roles of protein arginine methyltransferase in multiple myeloma. Mol. Ther. Oncol..

[15] Paik W.K., Kim S. (1968). Protein methylase I. Purification and properties of the enzyme. J. Biol. Chem..

[16] Kakimoto Y., Akazawa S. (1970). Isolation and identification of N-G,N-G- and N-G,N'-G-dimethyl-arginine, N-epsilon-mono-, di-, and trimethyllysine, and glucosylgalactosyl- and galactosyl-delta-hydroxylysine from human urine. J. Biol. Chem..

[17] Baldwin G.S., Carnegie P.R. (1971). Specific enzymic methylation of an arginine in the experimental allergic encephalomyelitis protein from human myelin. Science.

[18] Lin W.J., Gary J.D., Yang M.C., Clarke S., Herschman H.R. (1996). The mammalian immediate-early TIS21 protein and the leukemia-associated BTG1 protein interact with a protein-arginine N-methyltransferase. J. Biol. Chem..

[19] Henry M.F., Silver P.A. (1996). A novel methyltransferase (Hmt1p) modifies poly(A)+-RNA-binding proteins. Mol. Cell Biol..

[20] Bedford M.T., Frankel A., Yaffe M.B., Clarke S., Leder P., Richard S. (2000). Arginine methylation inhibits the binding of proline-rich ligands to Src homology 3, but not WW, domains. J. Biol. Chem..

[21] Chen D., Ma H., Hong H., Koh S.S., Huang S.M., Schurter B.T. (1999). Regulation of transcription by a protein methyltransferase. Science.

[22] Wang H., Huang Z.Q., Xia L., Feng Q., Erdjument-Bromage H., Strahl B.D. (2001). Methylation of histone H4 at arginine 3 facilitating transcriptional activation by nuclear hormone receptor. Science.

[23] Pal S., Vishwanath S.N., Erdjument-Bromage H., Tempst P., Sif S. (2004). Human SWI/SNF-associated PRMT5 methylates histone H3 arginine 8 and negatively regulates expression of ST7 and NM23 tumor suppressor genes. Mol. Cell Biol..

[24] Feng Y., Maity R., Whitelegge J.P., Hadjikyriacou A., Li Z., Zurita-Lopez C. (2013). Mammalian protein arginine methyltransferase 7 (PRMT7) specifically targets RXR sites in lysine- and arginine-rich regions. J. Biol. Chem..

[25] Zurita-Lopez C.I., Sandberg T., Kelly R., Clarke S.G. (2012). Human protein arginine methyltransferase 7 (PRMT7) is a type III enzyme forming omega-NG-monomethylated arginine residues. J. Biol. Chem..

[26] Boisvert F.M., Côté J., Boulanger M.C., Richard S. (2003). A proteomic analysis of arginine-methylated protein complexes. Mol. Cell Proteomics.

[27] Boisvert F.-M., Coté J., Boulanger M.-C., Cléroux P., Bachand F., Autexier C. (2002). Symmetrical dimethylarginine methylation is required for the localization of SMN in cajal bodies and pre-mRNA splicing. J. Cell Biol..

[28] Friesen W.J., Massenet S., Paushkin S., Wyce A., Dreyfuss G. (2001). SMN, the product of the spinal muscular atrophy gene, binds preferentially to dimethylarginine-containing protein targets. Mol. cell.

[29] Brahms H., Meheus L., de Brabandere V., Fischer U., Luhrmann R. (2001). Symmetrical dimethylation of arginine residues in spliceosomal Sm protein B/B' and the Sm-like protein LSm4, and their interaction with the SMN protein. RNA.

[30] Yang Y., Lu Y., Espejo A., Wu J., Xu W., Liang S. (2010). TDRD3 is an effector molecule for arginine-methylated histone marks. Mol. cell.

[31] Tripsianes K., Madl T., Machyna M., Fessas D., Englbrecht C., Fischer U. (2011). Structural basis for dimethylarginine recognition by the tudor domains of human SMN and SPF30 proteins. Nat. Struct. Mol. Biol..

[32] Liu K., Guo Y., Liu H., Bian C., Lam R., Liu Y. (2012). Crystal structure of TDRD3 and methyl-arginine binding characterization of TDRD3, SMN and SPF30. PLoS One.

[33] Brahms H., Raymackers J., Union A., de Keyser F., Meheus L., Luhrmann R. (2000). The C-terminal RG dipeptide repeats of the spliceosomal Sm proteins D1 and D3 contain symmetrical dimethylarginines, which form a major B-cell epitope for anti-Sm autoantibodies. J. Biol. Chem..

[34] Chan-Penebre E., Kuplast K.G., Majer C.R., Boriack-Sjodin P.A., Wigle T.J., Johnston L.D. (2015). A selective inhibitor of PRMT5 with in vivo and in vitro potency in MCL models. Nat. Chem. Biol..

[35] Cheng D., Yadav N., King R.W., Swanson M.S., Weinstein E.J., Bedford M.T. (2004). Small molecule regulators of protein arginine methyltransferases. J. Biol. Chem..

[36] Mavrakis K.J., McDonald E.R., Schlabach M.R., Billy E., Hoffman G.R. (2016). Disordered methionine metabolism in MTAP/CDKN2A-deleted cancers leads to dependence on PRMT5. Science.

[37] Kryukov G.V., Wilson F.H., Ruth J.R., Paulk J., Tsherniak A., Marlow S.E. (2016). MTAP deletion confers enhanced dependency on the PRMT5 arginine methyltransferase in cancer cells. Science.

[38] Marjon K., Cameron M.J., Quang P., Clasquin M.F., Mandley E., Kunii K. (2016). MTAP deletions in cancer create vulnerability to targeting of the MAT2A/PRMT5/RIOK1 axis. Cell Rep..

[39] Wang Y., Zhou J., He W., Fu R., Shi L., Dang N.K. (2024). SART3 reads methylarginine-marked glycine- and arginine-rich motifs. Cell Rep..

[40] Engstrom L.D., Aranda R., Waters L., Moya K., Bowcut V., Vegar L. (2023). MRTX1719 is an MTA-cooperative PRMT5 inhibitor that exhibits synthetic lethality in preclinical models and patients with MTAP-deleted cancer. Cancer discov..

[41] Smith C.R., Aranda R., Bobinski T.P., Briere D.M., Burns A.C., Christensen J.G. (2022). Fragment-based discovery of MRTX1719, a synthetic lethal inhibitor of the PRMT5•MTA complex for the treatment of MTAP-deleted cancers. J. Med. Chem..

